# Circulating miRNAs as biomarkers in diffuse large B-cell lymphoma: a systematic review

**DOI:** 10.18632/oncotarget.25230

**Published:** 2018-04-27

**Authors:** Maria Lopez-Santillan, Ane Larrabeiti-Etxebarria, Javier Arzuaga-Mendez, Elixabet Lopez-Lopez, Africa Garcia-Orad

**Affiliations:** ^1^ Department of Genetics, Physical Anthropology and Animal Physiology, Faculty of Medicine and Nursery, University of The Basque Country (UPV/EHU), Leioa, Spain; ^2^ Medical Oncology Service, Basurto University Hospital, Bilbao, Spain; ^3^ Pharmacy Service, Araba University Hospital-Txagorritxu, Vitoria, Spain; ^4^ Hematology and Hemotherapy Service, Cruces University Hospital, Barakaldo, Spain; ^5^ BioCruces Health Research Institute, Barakaldo, Spain

**Keywords:** lymphoma, circulating microRNA, diagnosis, classification, prognosis

## Abstract

Diffuse large B-cell lymphoma (DLBCL) is an aggressive and heterogeneous malignancy, with highly variable outcomes among patients. Although classification and prognostic tools have been developed, standard therapy still fails in 30-40% of patients. Hence, identification of novel biomarkers is needed. Recently, circulating microRNAs (miRNAs) have been suggested as non-invasive biomarkers in cancer. Our aim was to review the potential role of circulating miRNAs as biomarkers for diagnosis, classification, prognosis, and treatment response in DLBCL.

We performed a search in PubMed using the terms [((‘Non-coding RNA’) OR (‘microRNA’ OR ‘miRNA’ OR ‘miR’) OR (‘exosome’) OR (‘extracellular vesicle’) OR (‘secretome’)) AND (‘Diffuse large B cell lymphoma’ OR ‘DLBCL’)] to identify articles that evaluated the impact of circulating miRNAs as diagnosis, subtype, treatment response or prognosis biomarkers in DLBCL in human population.

Among the twelve articles that met the inclusion criteria, eleven considered circulating miRNAs as biomarkers for diagnosis, two for classification, and five for prognosis or treatment response. The limited number of studies performed and lack of consistency in results make it difficult to draw conclusions about the role of circulating miRNAs as non-invasive biomarkers in DLBCL. Although the preliminary associations observed seem promising, the only consistent result is the upregulation of mir-21 in DLBCL patients, which could be a biomarker for diagnosis. Further studies are needed.

## INTRODUCTION

Diffuse large B-cell lymphoma (DLBCL) is the most common subtype of non-Hodgkin lymphoma (NHL), accounting for 30% to 40% of all newly diagnosed cases [1]. DLBCL is an aggressive malignancy, very heterogeneous in genetic abnormalities, clinical features and response to treatment. This heterogeneity results in highly variable outcomes among patients [2].

Combined chemotherapy of rituximab, cyclophosphamide, doxorubicin, vincristine, and prednisone (R-CHOP) is considered as the standard first line therapy for DLBCL. The application of R-CHOP has led to complete remission for 75-80% of patients [3]. However, 30-40% of patients that achieve complete remission relapse at a later stage and some patients have refractory disease. Alarmingly, those patients tend to respond poorly to additional chemotherapy lines [4].

In order to predict the outcome of patients with DLBCL, different tools have been developed. For instance, the International Prognostic Index (IPI) identifies four risk groups with predicted five-year survival ranging from 26% to 73% using different clinical factors (age, stage, serum LDH, ECOG performance status and number of extranodal sites) [5]. Nevertheless, some patients present an unfavorable course of disease despite a good prognostic index. Another molecular prognostic tool uses gene expression profiling (GEP) and immunohistochemical analysis to discriminate two molecular subtypes with different clinical outcomes independent of IPI stratification: the germinal center B-cell-like (GCB) DLBCL, and the activated B-cell-like (ABC) DLBCL, with 5-year survival rates of 60% and 35% respectively [6, 7]. However, these tools are not able to identify all the patients that will not respond to therapy and, hence, novel biomarkers to enable a better prognostic stratification are needed.

During the past decades, several novel molecular and biological candidates with diagnostic, predictive and prognostic potential in DLBCL have been suggested, including microRNAs (miRNAs). MiRNAs are small, regulatory, non-coding RNAs that can negatively regulate gene expression at the post-transcriptional level by binding to the 3´ untranslated region (UTR) of a target mRNA and leading to increased degradation or inhibition of translation [8]. Nowadays, hundreds of miRNAs have been identified as biomarkers in cancer [9–11]. Interestingly, recent evidence has emerged showing that tumor-associated miRNAs can also be detected in body fluids such as serum or plasma [12]. This is possible thanks to the fact that miRNAs are protected from degradation in serum or plasma through association with RNA-binding proteins and/or packaging inside extracellular vesicles [13], providing them with high stability. This characteristic makes it possible to use non-invasive techniques for their evaluation, which is a great advantage regarding disease monitoring. Therefore, circulating miRNAs have emerged as candidate non-invasive biomarkers for diagnosis, and prognosis in cancer [14–17].

To date, some groups have investigated the potential role of circulating microRNAs in DLBCL [18–28]. Nevertheless, these studies present controversial results. We considered that it would be of interest to clarify these discrepancies. Therefore, herein, we review the potential role of circulating miRNAs as biomarkers for diagnosis, subtype characterization, prediction of treatment response and prognosis in patients with DLBCL.

## RESULTS

**Figure 1:**
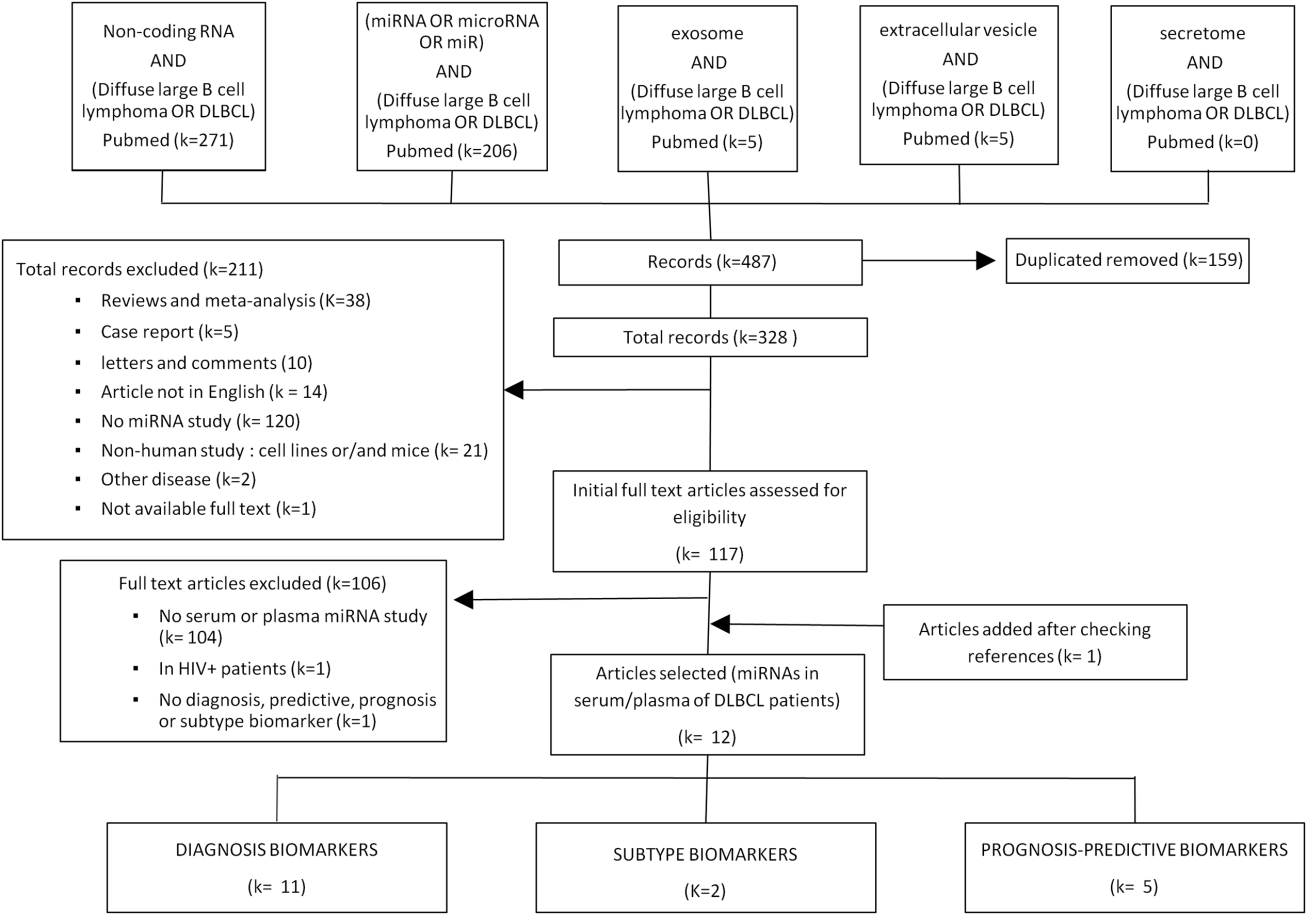
Flowchart of study selection

A total of 487 records were initially identified (Figure 1). Among them, 328 records remained after duplicates were removed. Among these records, 211 were discarded after abstract review because they clearly did not meet the required inclusion criteria. The full texts of the remaining 117 studies, which studied miRNAs in human DLBCL, were examined in detail. Further 104 articles were excluded because they were focused in tumor tissue and did not include circulating miRNAs, were focused in specific groups of patients with additional pathologies, or did not analyze microRNAs as diagnosis, subtype, treatment response, or prognosis biomarkers. After reviewing the references of the identified articles, one additional study was included. Finally, 12 articles investigating the role of circulating miRNAs as biomarkers in DLBCL were included. Eleven of them considered miRNAs as putative DLBCL diagnosis biomarkers, two articles searched for markers for subtype classification, and five articles considered treatment response or prognosis as their endpoint.

### Circulating miRNAs as non-invasive biomarkers for diagnosis in DLBCL

Eleven articles searched for circulating miRNAs deregulated in DLBCL patients vs. healthy controls (Table 1) [18–20, 22–29]. Those deregulated miRNAs could be used as biomarkers for diagnosis in this malignancy.

**Table 1:** Circulating miRNAs as non-invasive biomarkers for diagnosis in DLBCL

miRNAs	Result	Sample	miRNA source (method)	Method	Reference
159 miRNAs miR-124 miR-532-5p miR-122 miR-128 miR-141 miR-145 miR-197 miR-345 miR-424 miR-425 miR-101 miR-324 let-7e miR-222 miR-29c miR-375 miR-324-5p miR-135a miR-379 let-7i^*^ miR-32 Other	upregulation upregulation downregulation downregulation downregulation downregulation downregulation downregulation downregulation downregulation downregulation downregulation downregulation downregulation downregulation downregulation downregulation downregulation downregulation downregulation downregulation NS	14 DLBCL vs. 20 controls	Plasma/plasma exosomes (RNeasy MinElute)	Illumina HISeq sequencing	Khare D, et al (2017) [28]
2588 miRNAs miR-34a-5p miR-323b-3p miR-431-5p	51 DEMs upregulation downregulation downregulation	3 DLBCL vs. 3 controls	Serum (miRcute, Tiangen)	Illumina HISeq sequencing and qRT-PCR (SYBR Green)	Meng Y, et al (2017) [27]
miR-21	upregulation	203 DLBCL vs. 100 controls	Serum (miRNeasy Serum/Plasma)	qRT-PCR (miScript SYBR Green)	Zheng Z, et al. (2017) [26]
miR-155	NS	5 DLBCL vs. 18 controls	Serum extracellular vesicles (mirVana PARIS)	qRT-PCR (TaqMan)	Caivano A, et al. (2017) [25]
miR-21 miR-125b miR-130a miR-155 miR-200c miR-29c miR-145 miR-451	upregulation upregulation upregulation upregulation upregulation downregulation downregulation downregulation	56 DLBCL vs. 20 controls	Serum (miRcute, Tiangen)	qRT-PCR (miRcute, Tiangen).	Yuan WX, et al. (2016) [24]
miR-21	upregulation	112 DLBCL vs. 45 controls	Serum (Trizol)	qRT-PCR (TaqMan microRNA)	Li J, et al (2015) [23]
miR-17 miR-20b miR-210 miR-296	NS NS NS NS	21 DLBCL vs. 6 controls	Serum (miRNeasy Serum/Plasma)	qRT-PCR (TaqMan Small RNA)	Borges NM, et al. (2016) [22]
miR-15a-3p miR-21-5p miR-210-5p miR-181a-5p miR-155-5p miR-210-3p	upregulation upregulation upregulation downregulation NS NS	33 DLBCL vs. 22 controls	Serum (mirVana PARIS)	qRT-PCR (TaqMan microRNA)	Inada, et al (2015) [29]
miR-21	upregulation	62 DLBCL vs. 50 controls	Serum (mirVana)	qRT-PCR (TaqMan microRNA)	Chen W, et al (2014) [20]
miR-15a miR-16-1 miR-29c miR-155 miR-34a miR-21 miR-223	upregulation upregulation upregulation upregulation downregulation NS NS^*^	75 DLBCL vs. 77 controls	Serum (Trizol)	qRT-PCR (SYBR Green)	Fang C, et al (2012) [19]
miR-21 miR-155 miR-210	upregulation upregulation upregulation	60 DLBCL vs. 43 controls	Serum (Trizol)	qRT-PCR (Taqman microRNA)	Lawrie CH, et al. (2008) [18]

upregulation: Significantly increased expression in DLBCL patients.

downregulation: Significantly decreased expression in DLBCL patients.

NS: no significant difference in expression between patients and controls.

DEM: differentially expressed miRNAs.

^*^: analyzed in a subset of 20 patients vs. 20 controls.

Considering common miRNAs analyzed across different studies, we only identified seven miRNAs found to be significantly deregulated in DLBCL patients in at least two studies (miR-15a, miR-21, miR-29c, miR-34a, miR-145, miR-155, and miR-210) (Table 2) [18–20, 22–29]. Among them, five miRNAs showed conflicting results. On the one hand, miR-29c and miR-34a were found contradictorily upregulated or downregulated in DLBCL patients in different studies. On the other hand, miR-15a, miR-155 and miR-210 were found upregulated or unchanged in patients in the different studies in which they were analyzed.

**Table 2:** Circulating miRNAs explored by 2 or more studies as non-invasive biomarkers for diagnosis in DLBCL

miRNAs	upregulation	downregulation	NS
miR-15a	[19, 29]		[28]
miR-21	[18, 20, 23, 24, 26, 29]		[19, 28]
miR-29c	[19]	[24, 28]	
miR-34a	[27]	[19]	[28]
miR-145		[24, 28]	
miR-155	[18, 19, 24]		[25, 29]
miR-210	[18, 29]		[22, 28, 29]

upregulation: Significantly increased expression in DLBCL patients.

downregulation: Significantly decreased expression in DLBCL patients.

NS: no significant difference in expression between patients and controls.

Promisingly, other two miRNAs showed more homogeneous results. For instance, miR-145 was consistently downregulated in DLBCL patients. However, it must be noted that this result was only supported by the two studies in which it was studied [24, 28]. Interestingly, miR-21 was found accordingly upregulated in six of the eight studies in which it was analyzed [18, 20, 23, 24, 26, 29], pointing to this miRNA as a potential diagnosis biomarker in DLBCL.

### Circulating miRNAs as non-invasive biomarkers for subtype classification in DLBCL

The potential role of circulating microRNAs in DLBCL classification has been analyzed in a limited set of only two studies (Table 3) [20, 21]. A total of six microRNAs were studied comparing patients with ABC and GCB subtypes to search for differences among them. Each miRNA was analyzed in only one study. Among them, only miR-21 was found to be differentially expressed between subtypes, being upregulated in the subgroup of patients with ABC subtype [20].

**Table 3:** Circulating miRNAs as non-invasive biomarkers for ABC/GCB subtype classification in DLBCL

miRNAs	Result	Sample	miRNA source	Method	Reference
miR-33a miR-224 miR-455-3p miR520d-3p miR-1236	NS NS NS NS NS	173 DLBCL (81 ABC vs 92 GCB)	Serum (Trizol)	qRT-PCR (TaqMan microRNA)	Song G, et al. (2014) [21]
miR-21	upregulation	62 DLBCL (30 ABC vs 32 GCB)	Serum (mirVana)	qRT-PCR (TaqMan microRNA)	Chen W, et al (2014) [20]

upregulation: Significantly increased expression in ABC subtype.

NS: no significant difference in expression between subtypes.

### Circulating miRNAs as non-invasive biomarkers for prediction of response to R-CHOP therapy in DLBCL

Only two studies have focused in circulating miRNAs as predictive biomarkers of response to R-CHOP treatment in DLBCL patients [21, 24] (Table 4).

**Table 4:** Circulating miRNAs as non-invasive biomarkers for prediction of of response to R-CHOP therapy in DLBCL

miRNAs	Result	Sample	miRNA source	Method	Reference
miR-125b miR-130a miR-21 miR-29c miR-145 miR-155 miR-200c miR-451	upregulation upregulation NS^*^ NS^*^ NS^*^ NS^*^ NS^*^ NS^*^	56 DLBCL (21 resistant vs 35 sensitive)	Serum (miRcute, Tiangen)	qRT-PCR (miRcute, Tiangen).	Yuan WX, et al. (2016) [24]
736 miRNAs miR-224 miR-520d-3p miR-1236 miR-33a miR-455-3p Other^**^	upregulation upregulation upregulation downregulation downregulation NS	133 DLBCL	Serum (Trizol)	qRT-PCR (TaqMan microRNA)	Song G, et al. (2014) [21]

upregulation: Significantly increased expression in drug resistant DLBCL patients.

downregulation: Significantly decreased expression in drug resistant DLBCL patients.

NS: no significant difference in expression between resistant and sensitive patients.

^*^Studied in a subgroup of 20 DLBCL (10 chemo-resistant vs 10 chemo-sensitive).

^**^Studied in a subgroup of 40 DLBCL (20 chemo-resistant vs 20 chemo-sensitive).

The first study analyzed 736 miRNAs in serum samples of 20 complete remission and 20 primarily refractory DLBCL patients. Five miRNAs were differentially expressed between both groups (miR-224, miR-1236, miR-520d-3p, miR-33a, and miR-455-3p) and were validated in an independent group of 133 patients. Upregulation of miR-455-3p and miR-33a was found to be associated with chemosensitivity while upregulation of miR-224, miR-1236, and miR-520d-3p was associated with chemoresistance [21].

The second study analyzed a group of eight miRNAs (miR-21, miR-29, miR-125b, miR-130a, miR-145, miR-155, miR-200c and miR-451) finding the upregulation of miR-125b and miR-130a to be associated with resistance to R-CHOP treatment [24].

MiR-21 was the only miRNA analyzed in both studies. Nevertheless, its expression was not significantly associated with treatment response in any of the two studies.

### Circulating miRNAs as non-invasive biomarkers for prognosis prediction in DLBCL

Finally, the relevance of circulating microRNAs for prognosis prediction in DLBCL patients was considered in five studies [18, 20, 21, 23, 24] (Table 5). A total of ten microRNAs were analyzed. Among them, only miR-21 was studied in more than one article, providing discordant results. Upregulated miR-21 has been described as an independent poor prognostic factor in one of the studies [23]. However, the other two studies [18, 20] have shown its upregulation associated with good prognosis. Regarding the other miRNAs, which were analyzed each of them by an only study, upregulation of miR-125b was found to be associated with poor prognosis in patients with DLBCL [24]. On the other hand, high expression of miR-224, miR-1236, and miR-520d-3p and low expression of miR-455-3p and miR-33a were found to be individually associated with unfavorable outcome and a score based on this five-miRNAs was proposed to predict the clinical outcome of DLBCL patients treated with R-CHOP regimen, independent from the IPI score [21].

**Table 5:** Circulating miRNAs as non-invasive biomarkers for prognosis prediction in DLBCL

miRNAs	Result	Outcome	Sample	miRNA source	Method	Reference
miR-125b miR-130a	- NS	OS	56 DLBCL	Serum (miRcute, Tiangen)	qRT-PCR (miRcute, Tiangen).	Yuan WX, et al. (2016) [24]
miR-21	-	OS	112 DLBCL	Serum (Trizol)	qRT-PCR (TaqMan microRNA)	Li J, et al (2015) [23]
miR-33a miR-455-3p miR-224 miR-520d-3p miR-1236	+ + - _ _	MRT/PR	133 DLBCL	Serum (Trizol)	qRT-PCR (TaqMan microRNA)	Song G, et al. (2014) [21]
miR-21	+	RFS	62 DLBCL	Serum (mirVana)	qRT-PCR (TaqMan microRNA)	Chen W, et al (2014) [20]
miR-21 miR-155 miR-210	+^*^ NS NS	RFS / OS	52 DLBCL	Serum (Trizol)	qRT-PCR (Taqman microRNA)	Lawrie CH, et al. (2008) [18]

OS: overall survival; MRT: median remission time; PR: probability of remission; PFS: progression free survival; RFS: relapse free survival; -: high expression of the miRNA significantly associated with worse outcome; +: high expression of the miRNA significantly associated with better outcome; ^*^ statistically significant association with better RFS but no significant association with OS.

## DISCUSSION

In this systematic review, we have performed a deep analysis of the current literature in relation to the potential role of circulating miRNAs as non-invasive biomarkers for diagnosis, subtype characterization, treatment response and prognosis in patients with DLBCL.

Regarding the suitability of circulating miRNAs as diagnostic biomarkers in DLBCL, eleven articles were identified, in which a total of seven miRNAs (miR-15a, miR-29c, miR-34a, miR-155 and miR-210) were found at least twice to be significantly deregulated in DLBCL patients [18–20, 22–29]. Among them, only miR-145 and miR-21 presented concordant results. Contradictory results could be due to different factors, including differences in methodology, or miRNA source, though in these studies those factors are quite homogeneous. On the other hand, it must be noted that most studies were performed with limited sample sizes and statistical power. Consequently, further larger studies are needed to clarify the results.

MiR-145, which was downregulated in DLBCL patients in the two studies in which it was analyzed [24, 28], is a tumor-suppressor miRNA that has been found to be downregulated in several cancer types, i.e. colorectal cancer, bladder cancer, or ovarian cancer [30–32]. In fact, some of the validated targets of this miRNA include known hallmarks of DLBCL, such as *c-MYC* or *ETS1* [33–39]. However, such a limited number of studies with modest sample sizes does not allow to reach final conclusions. Further studies would be required in order to validate the putative role of mir-145 as a non-invasive biomarker for diagnosis in DLBCL.

On the other hand, it is noteworthy that miR-21, which was analyzed in eight independent studies, was significantly upregulated in DLBCL patients in six of them [18, 20, 23, 24, 26, 29]. In agreement with this observation, miR-21 is one of the most frequently up-regulated miRNAs in solid tumors and high levels are also observed in B-NHLs, being considered an oncomiR. In fact, overexpression of miR21 experimentally leads to a pre-B malignant lymphoid-like phenotype [40]. In addition, miR-21 has been described as a key regulator of disease progression in B-cell lymphoma [41]. Its role in tumorigenesis and disease progression has been associated to the inhibition of the expression of phosphatases, limiting the activity of signaling pathways such as PI3K/AKT, recently suggested to play a crucial role in mediating growth, proliferation and cell survival in a substantial number of DLBCL patients [42, 43]. In summary, the biological role of miR-21 and its replicated upregulation in circulating fluids suggest that miR-21 could be a good biomarker for DLBCL diagnosis.

The utility of circulating microRNAs for DLBCL classification has been analyzed by only two studies with no coincidence in the miRNAs considered [20, 21]. Only miR-21 was found to be upregulated in the ABC subgroup, which is associated with lower survival, in a subset of 62 DLBCL patients [20]. Since this association was observed in only one study with a limited sample size, it would need to be further explored by additional research.

Focusing on circulating miRNAs as predictive biomarkers of response to R-CHOP treatment, two studies were identified [21, 24]. The only miRNA analyzed in both investigations, mir-21, was not associated with treatment response in any of the studies. Interestingly, among the 736 miRNAs analyzed in the first study, upregulation of miR-455-3p and miR-33a was associated with chemosensitivity while upregulation of miR-224, miR-1236, and miR-520d-3p was associated with chemoresistance [21]. In the second study, eight miRNAs were analyzed, finding the upregulation of miR-125b and miR-130a to be associated with R-CHOP chemoresistance [24]. These results would need to be confirmed in additional studies.

Finally, the implication of circulating microRNAs in prognosis in DLBCL has been analyzed in five studies including ten miRNAs [18, 20, 21, 23, 24]. Among them, only miR-21 has been studied in more than one study. However, the results for this miRNA are contradictory. On the one hand, a multivariate analysis, showed upregulated miR-21 as an independent poor prognostic factor [23]. On the contrary, the other two studies that considered this miRNA have found its upregulation to be associated with good prognosis [18, 20]. Surprisingly, in the first of these studies, upregulation of miR-21 was also associated with ABC-DLBCL subgroup, which usually behaves more aggressively and is associated with worse outcome [20]. A possible explanation for this apparently contradictory situation is that serum miR-21 expression was higher in patients with DLBCL stage I and II in comparison to those with III and IV, which could be a confounding factor. On the other hand, Lawrie et al also showed miR-21 upregulation associated with good prognosis [18]. In this case, even though no multivariate analysis was performed, no association between microRNA levels and clinicopathological features was observed (i.e. sex, IPI, stage and presence of extranodal disease). As a result, the significance of miR-21 in prognosis of DLBCL remains uncertain.

Focusing in miRNAs analyzed by a single study, upregulation of miR-125b has been associated with poor prognosis [24]. In addition, a predictor score based on a signature of five miRNAs, among which high expression of miR-224, miR-1236, and miR-520d-3p and low expression of miR-455-3p and miR-33a were individually associated with unfavorable outcome, has been proposed to predict the clinical outcome of DLBCL patients, independent from the IPI score [21]. However, all these results need to be confirmed, given the limited evidence provided.

In brief, the limited number of studies performed, which usually consider a limited set of miRNAs, and the lack of consistency in the results obtained make it difficult to draw final conclusions about the role of circulating miRNAs as non-invasive biomarkers in DLBCL. Although the preliminary associations observed could be of interest, the only result that seems sounder so far is the upregulation of mir-21 in DLBCL patients, which could be used for diagnosis. Consequently, it would be of particular interest to perform large-scale studies including larger sample sizes and a wider array of miRNAs. We can conclude that even if this is a very promising field of study, published evidence is very limited and further studies are needed.

## MATERIALS AND METHODS

A systematic search in PubMed database was performed to identify articles published between November 1975 and November 2017 using the following strategy: [((‘Non-coding RNA’) OR (‘microRNA’ OR ‘miRNAs’ OR ‘miR’) OR (‘exosome’) OR (‘extracellular vesicle’) OR (‘secretome’)) AND (‘Diffuse large B cell lymphoma’ OR ‘DLBCL’)].

Articles were included if they presented independent original studies and evaluated the impact of circulating miRNAs as diagnosis, subtype, treatment response or prognosis biomarkers in DLBCL in human population. Reviews and meta-analyses, case reports, letters, comments, abstracts, and articles not published in English were not included. Studies were also excluded if they did not analyze miRNAs, did not include data from human populations, or were focused on other diseases. After full text assessment, articles that did not analyze circulating miRNAs, included other diseases, or did not assess the role of miRNAs in diagnosis, subtype, treatment response, or prognosis, were excluded. All references within the identified studies were then reviewed in order to identify additional matches.

Each eligible manuscript was assessed independently by two researchers (ML and AL). Disagreements were resolved by consensus. Data extracted from each study included: publication year, type of blood-based fluid analyzed (serum or plasma), characteristics of the study population (Table 6), technical methodology, miRNAs assessed, and the list of specific miRNAs that could be used as biomarkers in DLBCL. In order to define associations between the expression of the miRNAs and the phenotypes, a p value < 0.05 was considered statistically significant.

**Table 6:** Clinical characteristics of the study population in each included study

Study	Sample	Age	Sex (%)		Hans’ algorithm (%)			Performance status (%)		Ann Arbor staging (%)				IPI (%)	
		Median (range) or mean ± SD	Female	Male	GCB	non-GCB	nap	PS 0-2	PS 3-4	I	II	III	IV	0-2	3-5
Khare D, et al (2017) [28]	14 DLBCL	64 (34-85)	36	64	NA	NA	NA	NA	NA	NA	NA	NA	NA	50	50
	20 controls	37 (26-63)	45	55											
Meng Y, et al (2017) [27]	3 DLBCL	NA	NA	NA	NA	NA	NA	NA	NA	NA	NA	NA	NA	NA	NA
	3 controls	NA	NA	NA											
Zheng Z, et al. (2017) [26]	203 DLBCL	NA	39.4	60.6	NA	NA	NA	NA	NA	56.7		43.3		66	34
	100 controls	NA	NA	NA											
Caivano A, et al. (2017) [25]	5 DLBCL	66 (53–80)	60	40	NA	NA	NA	NA	NA	NA	NA	NA	NA	NA	NA
	18 controls	60 (43–77)	55.6	44.4											
Yuan WX, et al. (2016) [24]	56 DLBCL	54.7 (23-74)	43	57	NA	NA	NA	NA	NA	10	24	38	28	28	72
	20 controls	NA	NA	NA											
Li J, et al (2015) [23]	112 DLBCL	NA	48.2	51.8	NA	NA	NA	NA	NA	42		58		37.5	62.5
	45 controls	NA	NA	NA											
Borges NM, et al. (2016) [22]	21 DLBCL	NA	NA	NA	NA	NA	NA	NA	NA	NA	NA	NA	NA	NA	NA
	6 controls	NA	NA	NA											
Inada, et al (2015) [29]	33 DLBCL	67 (36-84)	39	61	42.4	48.5	9.1	91	9	9.1	21.2	18.2	51.5	55.6	45.4
	22 controls	62 (20-76)	41	59											
Chen W, et al (2014) [20]	62 DLBCL	66.1 ±13.6	33.8	66.1	51.6	48.4	0	NA	NA	30.7	17.7	19.3	32.3	71	29
	50 controls	NA	NA	NA											
Fang C, et al (2012) [19]	75 DLBCL	54 (19–85)	45.3	54.7	NA	NA	NA	NA	NA	13.3	16	22.7	48	60	40
	77 controls	50 (36–68)	41.5	58.4											
Lawrie CH, et al. (2008) [18]	60 DLBCL	63 ±11	35	65	NA	NA	NA	NA	NA	33	23	30	13	70	30
	43 controls	NA	NA	NA											
Song G, et al. (2014) (21)	173 DLBCL	56.4 (19-78)	47.4	52.6	53.2	46.8	0	85.5	14.5	14.5	25.4	35.3	24.8	22.3	67.7

NA: Data not available. Nap: unclassified.

## Abbreviations

DLBCL: Diffuse large B-cell lymphoma
miRNAs: microRNAs
R-CHOP: Combined chemotherapy of rituximab, cyclophosphamide
IPI: International Prognostic Index
GEP: gene expression profiling
GCB: center B-cell-like DLBCL
ABC: activated B-cell-like DLBCL
UTR: untranslated region
